# Investigation into the Structure and Properties of Biochar Co-Activated by ZnCl_2_ and NaHCO_3_ under Low Temperature Conditions

**DOI:** 10.3390/ma17040942

**Published:** 2024-02-18

**Authors:** Hao Zhang, Zhaozhou Wei, Deyuan Xiong, Yao Wu, Menglong Tong, Huiping Su, Zuoyuan Zhang, Jian Liao

**Affiliations:** 1 School of Chemistry and Chemical Engineering, Guangxi University, Nanning 530004, China; zhwjszs@163.com (H.Z.); zzwei0608@outlook.com (Z.W.); 2014302069@st.gxu.edu.cn (Y.W.);; 2 Guangxi Key Laboratory of Petrochemical Resource Processing and Process Intensification Technology, School of Chemistry and Chemical Engineering, Guangxi University, Nanning 530004, China

**Keywords:** hierarchically porous carbons, biomass, chemical activation, solid acid catalyst

## Abstract

Using sodium lignosulfonate as feedstock, ZnCl_2_ and NaHCO_3_ co-activated the hierarchical porous carbons (HPCs) were prepared by one-pot pyrolysis with different NaHCO_3_ dosages (0–4 g) and carbonization temperatures (400–600 °C). Subsequently, phosphotungstate (HPW) was supported with the resulting biochar for the α-pinene hydration reaction to produce α-terpineol. The optimum preparation conditions were determined according to the yield of α-terpineol. The formation mechanism and physicochemical properties of HPCs were analyzed through TG, SEM, XPS, XRD, FT-IR, and N_2_ adsorption–desorption isotherms. The results demonstrated that NaHCO_3_ underwent a two-step reaction which liberated a substantial quantity of CO_2_, thereby enhancing activated carbon’s macroporous and mesoporous structures. Simultaneously, NaHCO_3_ mitigated strong acid gas (HCl) emissions during ZnCl_2_ activation. Compared with AC450-4:8:0 prepared by ZnCl_2_ activation alone, the total pore volume of AC450-4:8:2 prepared by co-activation is increased from 0.595 mL/g to 0.754 mL/g and the mesopore rate from 47.7% to 77.8%, which is conducive to reducing the steric hindrance of the hydration reaction and improving the selectivity. Hydration experiments show that the selectivity of α-terpineol is 55.7% under HPW/AC450-4:8:2 catalysis, higher than 31.0% for HPW and 47.4% for HPW/AC450-4:8:0.

## 1. Introduction

Activated carbon with a porous structure has received extensive attention from many scholars. It was widely used in many fields, such as wastewater treatment, CO_2_ adsorption, catalyst support, anaerobic digestion, energy storage, etc., because of its high specific surface area, adjustable porosity, good electrical conductivity, excellent stability, and low cost. Biomass became the primary precursor of activated carbon obtained by carbonization and activation due to its wide range of sources. During biomass pyrolysis, the dehydration process and the release of volatile components were conducive to developing primary pores and forming stable aromatic carbon structures [1]. Due to the limited pore-forming capacity of volatile components, activators played an important role in improving the pore structure of activated carbon.

Different activation methods produced pore structures with varying characteristics in the typical temperature range. The activation process was divided into physical activation and chemical activation. In physical activation, the carbon was etched at higher temperatures by oxidizing gases, including oxygen, water vapor, and carbon dioxide, to facilitate the development of micropores. According to Lee, J. H. et al., the specific surface area of activated carbon increased significantly (1409 m^2^/g to 2506 m^2^/g) with increasing temperature (900 °C to 1000 °C) and was mainly contributed by micropores [2]. In chemical activation, activation reagents with different properties were extensively reported, mainly including bases (KOH or NaOH), acids (H_3_PO_4_), and metal salts (ZnCl_2_, MgCl_2_, K_2_CO_2_, KHCO_3_, etc.). Among them, when KOH was used as the activator, many micropores and a few mesopores were obtained through the redox reaction of various potassium compounds with carbon, mainly occurring above 700 °C [3,4]. Illingworth, J. M et al. used flax fiber as a precursor for KOH activation at 800 °C, and the specific surface area of activated carbon was up to 1656 m^2^/g, which was mainly contributed by micropores [5]. Phosphoric acid was a commonly used acid activator, and the required activation temperature is usually only 350–500 °C. Hakan et al. reported that the specific surface area, total pore volume, and micropore volume of activated carbon reached 1455 m^2^/g, 0.88 cm^3^/g, and 0.66 cm^3^/g, respectively, after phosphoric acid activation at 400 °C [6]. The activation temperature of ZnCl_2_ was usually 400–600 °C, which is because, at lower temperatures, the biomass has not yet formed a stable polycondensation carbon structure, leading to imperfect pore structure, while at higher temperatures, a large amount of volatilization of ZnCl_2_ and the sintering effect of carbon lead to the reduction of pore area and volume [7,8,9,10]. Due to its swelling effect and template action, an appropriate amount of ZnCl_2_ could provide a rich microporous structure for the carbon at 400 °C. With the increase of ZnCl_2_ dosage and carbonization temperature, the activation was enhanced, and many volatile substances were released. The micropores began to transform into mesoporous pores [11,12]. Lin F et al. mixed solid ZnCl_2_ and buttonwood sawdust (2:1) in a fixed ratio to obtain activated carbon with a high specific surface area (S_BET_ = 2308 m^2^/g). As the temperature increased from 400 °C to 550 °C, the mesoporous volume percentage (V_meso_/V_Total_) increased from 30.4% to 58.6% [7]. The activation mechanism of carbonates and bicarbonates of Na and K above 800 °C was similar to that of KOH. Still, the difference was that bicarbonate decomposition at about 200 °C produces water vapor and carbon dioxide, resulting in the macroporous structure of activated carbon [13,14]. Therefore, bicarbonate activation yields hierarchical porous carbons (HPCs) with interconnected pore structures comprising macropores, mesoporous pores, and micropores [15]. Wang H et al. prepared HPCs with abundant pore structure from wheat straw by using KHCO_3_ as an activator, with a maximum S_BET_ of 1297 m^2^/g at 800 °C activation temperature [16]. In summary, sodium and potassium ions perform better in promoting the development of the internal microporous structure of activated carbon. Still, higher temperatures (≥700 °C) are required to provide sufficient activation energy. On the contrary, ZnCl_2_ and H_3_PO_4_ are favorable for the formation of micropores at lower temperatures. In addition, the role of bicarbonate in the development of macroporous structures is significant.

The application efficiency of HPCs with a hierarchical porous structure has been significantly enhanced in different fields, including mass transfer process, adsorption, charge storage, and so on [17]. However, pore sizes are often concentrated in narrow intervals under the action of a single activator. Therefore, scholars have designed different methods to prepare HPCs with rich pore structure, including the template, nanocasting, and activator combination methods. Marta Sevilla et al. used potassium oxalate as an activator and calcium carbonate as a rigid template to prepare HPCs above 700 °C [18]. Potassium oxalate is conducive to forming micropores, nitrogen in biomass precursors is conducive to developing mesoporous pores, and calcium carbonate creates conditions for constructing macropores. Federico Cesano et al. used KOH and ZnCl_2_ as activators, which promoted the formation of micropores and mesoporous pores at 800 °C, respectively [19]. Changli Qi et al. used poly (methyl methacrylate) as a carbon source and macropore template and SiO_2_ nanospheres as a mesoporous template to produce HPCs with relatively regular macropore interconnect network at 600 °C carbonization temperature [20]. Under high temperature conditions (≥600 °C), scholars have adopted different methods to prepare HPCs with abundant macro-, meso-, and micropores, but under lower temperatures (<600 °C), there are few studies. Low-temperature carbonization process has great research significance because of its advantages of low energy consumption, high carbon yield, and abundant oxygen-containing groups on the surface of carbon.

Lignin is the world’s second largest natural polymer after cellulose, with a carbon content of 60–65%, which can be used as a precursor for the preparation of activated carbon [21,22,23]. Lignosulfonate is produced through the sulfonation of lignin, mainly from sulfite pulp waste liquid, and is a by-product in the paper industry. The output is enormous (about 1 million tons of dry solids annually) [22]. Therefore, we choose sodium lignosulfonate (SL) as the precursor to prepare activated carbon to utilize biomass waste effectively. Therefore, based on the traditional ZnCl_2_ activation, this work introduced activator NaHCO_3_, which could provide abundant macroporous structure for carbons, and explored its influence on the micro-mesoporous structure of ZnCl_2_-activated carbon to obtain HPCs with productive microporous, mesoporous, and macroporous structures at the carbonization temperature of 400–600 °C. ZnCl_2_ can provide abundant micro-mesoporous structures in the low temperature range (400–600 °C). Finally, we used the prepared HPCs as a carrier loaded with phosphotungstic acid (HPW) for the reaction of α-pinene hydration to produce α-terpineol to explore the effect of the hierarchical porous structure on the catalytic performance of the catalyst.

## 2. Materials and Methods

### 2.1. Materials

Sodium lignosulfonate (SL), phosphotungstic acid (HPW), and ZnCl_2_ (98%) were purchased from McLean. α-pinene (98%) was obtained from Shanghai Aladdin Reagents (Shanghai, China). NaHCO_3_ (99.5%) and absolute ethyl alcohol (99.7%) purchased from Guangdong Guanghua Sci-Tech Co., LTD (Guangdong, China). CH_3_COCH_3_ (99.5%) was supplied from Chengdu Colon Chemical Co., LTD (Chengdu, China). All chemicals are used directly without purification.

### 2.2. Experimental Procedure

#### 2.2.1. Preparation of HPCs

The mass ratio of biomass to ZnCl_2_ was confirmed as 1:2, based on previous studies [7]. In a standard experiment, 4 g SL, 8 g ZnCl_2_, and a certain amount of NaHCO_3_ solids (0–4 g) were mixed evenly and transferred to a tube furnace with a nitrogen atmosphere. The heating was performed under nitrogen atmosphere flow (40 mL/min) at the rate of 10 °C/min at 400–600 °C for 2 h. After the thermal treatment, the samples were stirred in 1 M HCl solution at 100 °C for 1 min to remove metal compound impurities and cleaned with deionized water until neutral. After washing, the samples were dried at 105 °C for 24 h to obtain the HPCs (designated as ACT-a where “T” and “a” are the carbonization temperature and the mass ratio of SL, ZnCl_2_ and NaHCO_3_). In addition, activated carbon activated using only NaHCO_3_ was named AC450-4:0:2.

#### 2.2.2. The Load of HPW

The load of HPW was modified based on previous reports [24]. First, 1.0 g AC was added to a clear and transparent solution, which was obtained by dissolving 1.0 g phosphotungstic acid in 60 mL anhydrous ethanol. Subsequently, the suspension was rotated on a rotary evaporator to remove the solvent and dried overnight at 105 °C. Finally, the dried samples were calcined at 180 °C for 4 h. The pure HPW was subjected to calcination for utilization under identical conditions. The catalyst was designated HPW/ACT-a. The AC450-4:8:2 was physically blended with calcined HPW in a 1:1 mass ratio to prepare the control samples, denoted as AC450-4:8:2 (HPW).

#### 2.2.3. Hydration Reaction

α-pinene was hydrated to produce α-terpineol alcohols in a 100 mL round flask filled with 10.00 mL acetone, 2.50 mL α-pinene, 2.50 mL distilled water, and 0.25 g HPW/ACT-a. Under magnetic agitation, the substrate reacted in an oil bath at 80 °C for 24 h. After the reaction, acetone solvent, aqueous phase, and catalyst particle were successively removed to obtain an oil phase containing α-terpineol and other by-products by swirling evaporation, centrifugal separation, and filtration using 0.22 μm organic filter membrane. Finally, the oil phase was analyzed to determine the conversion and selectivity of the hydration reaction by GC. In order to investigate the efficacy of the solid acid catalyst synthesized in this study for the hydration reaction, four controlled experiments were conducted, including a blank group (without any catalyst added), AC450-4:8:2 (with only 0.125 g support added), HPW (with only 0.125 g of calcined HPW added), and AC450-4:8:2 (HPW) above. To investigate the recycling performance of the catalyst, the residual catalyst HPW/ACT-a after the reaction was collected, washed with acetone to remove the residual impurities and reactants, and reused in a subsequent hydration reaction under the same reaction conditions.

### 2.3. Products Analysis

Analytical method of gas chromatography is supplied in the Supplementary Material Text S1.

### 2.4. Characterization of the Solid Samples

Thermogravimetric analysis (TG) was performed on a thermogravimetric analyzer (TGA 550, TA Instruments, New Castle, DE, USA) under nitrogen atmosphere at a warming rate of 10 °C/min to investigate the pyrolytic properties of the samples. The morphology of the samples was observed by the scanning electron microscopy (SEM, TESCAN MIRA LMS, TESCAN, Brno, Czech Republic). N_2_ adsorption–desorption experiments were performed at Quantachrome Autosorb IQ3 (Anton Paar, Ashland, VA, USA) to obtain adsorption isotherms. The S_BET_ of HPC_s_ was calculated by the BET method, the average pore diameter (D_pore_) and total pore volume (V_Total_) were measured by the BJH method, micropore volume (V_micro_) was calculated by a t-plot equation, and the pore size distribution (PSD) was calculated by the DFT model. X-ray diffraction spectroscopy (XRD, Bruker D8 Advance, Karlsruhe, Germany), Raman scattering spectroscopy (WITec alpha300R, WITec GmbH, Ulm, Germany), Fourier transform infrared spectroscopy (FT-IR, Thermo Scientific iN10, Thermo Fisher Scientific, Waltham, MA, USA) and X-ray photoelectron spectroscopy (XPS, Thermo Scientific K-Alpha, Thermo Fisher Scientific, Waltham, MA, USA) were used to characterize the graphitization degree, surface functional groups, and elemental content of the samples. Energy dispersive X-ray (EDX) measurements of the catalyst were made with an EDS spectrometer (OXFORD Xplore, Oxford Instruments, Abingdon, UK) attached to the SEM (ZEISS GeminiSEM 300, ZEISS, Oberkochen, Germany).

Details of the acid-base titration experiment of the sample are found in the Supplementary Material Text S2.

## 3. Results

### 3.1. Formation Mechanism and Characterization of HPCs

#### 3.1.1. Thermogravimetric Analysis

**Figure 1:**
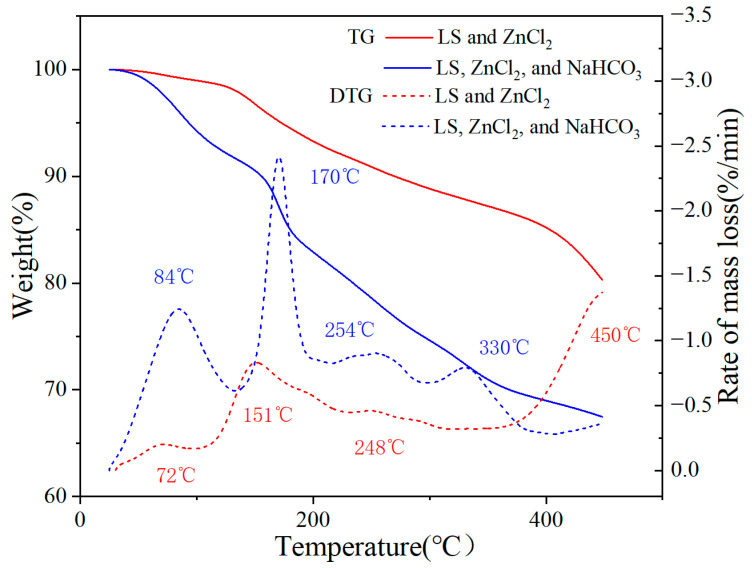
TG and DTG curves of SL and different activators (the mass ratios are 4:8:0 and 4:8:2, respectively).

Lignin releases a large number of volatile substances during heating. Existing studies have shown that ZnCl_2_ can significantly reduce the decomposition temperature of lignin from 441 °C to 242 °C [7]. Therefore, TG and DTG analyses were performed on mixtures containing ZnCl_2_ and SL to explore the effect of adding NaHCO_3_ on releasing volatiles during heating. The evaporation of water, the degradation of SL, and the decomposition of ZnCl_2_ occurred below 100 °C, around 150 °C, and above 400 °C, respectively [25,26]. Upon the addition of NaHCO_3_ (Figure 1), a significant decomposition peak appeared at 170 °C, attributed to NaHCO_3_ decomposition [13]. The appearance of a new decomposition peak at 330 °C, accompanied by the disappearance of the previous peak at 400–450 °C, could be attributed to the reaction between NaHCO_3_′s decomposition product Na_2_CO_3_ and unstable ZnCl_2_, resulting in a significant release of CO_2_ (Equation (3)). Additionally, distinct diffraction peaks corresponding to the wurtzite ZnO phase (JCPDS 36-1451) were observed in the XRD patterns (Figure S1) of solid samples obtained after carbonization of AC450-4:8:0 and AC450-4:8:2, confirming ZnO formation. A significant number of gas bubbles were generated during the pickling process of AC450-4:0:2, while no observable bubble formation was detected in AC450-4:8:2, confirming Na_2_CO_3_ consumption. The statement above served as the basis for reaction (1 and 3).

The macroscopic morphological characteristics (Figure S2) of the untreated samples after carbonization were observed and recorded using a camera after activation at different carbonization temperatures (250 °C, 350 °C, and 450 °C) under the weight ratio of SL, ZnCl_2_, and NaHCO_3_ (4:8:2). They were named AC250-4:8:2, AC350-4:8:2, and AC450-4:8:2. The AC350-4:8:2 exhibits the largest volume, which can be attributed to the significant release of volatile gases around 330 °C in GC analysis. In addition, AC450-4:0:2 (Figure S3a) showed a shrinking state, while AC450-4:8:2 (Figure S3b) had a limited expansion effect. Consequently, the “fermentation” process in the activation procedure was ascribed to the synergistic effect of ZnCl_2_ and NaHCO_3_. ZnCl_2_ acted as a template agent to prevent carbon collapse, while NaHCO_3_ served as a gas-producing reagent. This collaboration optimized the pore structure of HPCs.

ZnCl_2_ + H_2_O → ZnO + 2HCl (1)

2NaHCO_3_ → Na_2_CO_3_ + H_2_O + CO_2_ (2)

Na_2_CO_3_ + ZnCl_2_ → 2NaCl_2_ + ZnO + CO_2_ (3)

#### 3.1.2. Surface Morphology and Pore Characteristics Analysis

**Figure 2:**
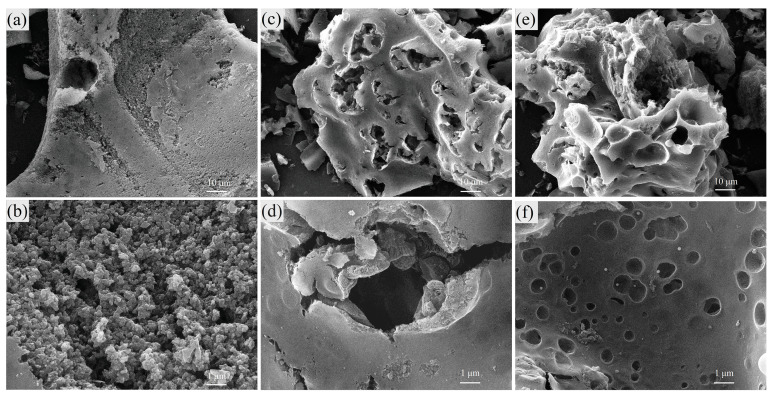
SEM images of AC450-4:8:0 (**a**,**b**); AC450-4:8:2 (**c**,**d**); AC450-4:8:4 (**e**,**f**).

The morphology of HPCs (Figure 2) was studied using scanning electron microscopy (SEM). The AC450-4:0:2 (Figure S3) exclusively exhibited a dense macropore structure consistent with the previous literature [16]. AC450-4:8:0 (Figure 2a,b) is a solid bulk material with few voids (>1 µm), featuring a rough surface and porous structure [27]. After adding the activator NaHCO_3_, the surface morphology of the carbon was significantly changed. AC450-4:8:2 (Figure 2c,d) and AC450-4:8:4 (Figure 2e,f) exhibited numerous voids and relatively smooth surfaces. This was attributed to the decomposition of NaHCO_3_ during heating, resulting in the release of a substantial amount of CO_2_ and water vapor. Consequently, this enhanced the carbon body’s pore structure and generated numerous convex voids. The rough surface of the void edge was attributed to ZnCl_2_ etching. Additionally, a series of concave holes appeared on the carbon surface, which was observed in the electron microscope image of AC450-4:8:4 (Figure 2f), and these holes were caused by alkali metal ions etching the carbon structure [14]. In conclusion, NaHCO_3_ addition enriched the pore structure of activated carbon on ZnCl_2_ activation, resulting in a hierarchical porous structure.

**Figure 3:**
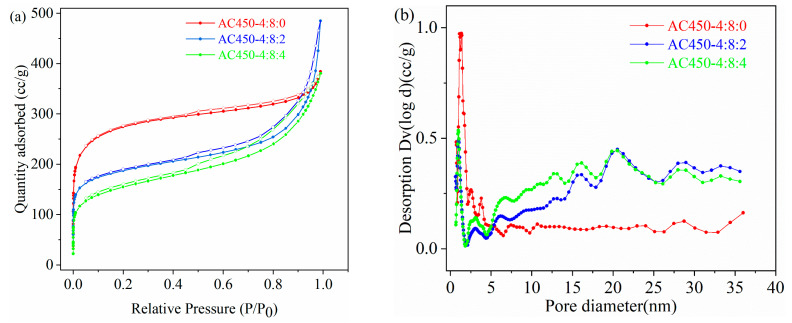
(**a**) N_2_ sorption isotherm and (**b**) pore size distribution curves of AC450-4:8:0, AC450-4:8:2, and AC450-4:8:4.

The SEM results showed that NaHCO_3_ significantly affects the surface morphology of activated carbon. The impact of NaHCO_3_ on the pore structure changes of activated carbon was detailed through N_2_ adsorption and desorption experiments. The adsorption capacity of AC450-4:8:0 increased rapidly at low relative pressure (P/P0 < 0.05) following a type I adsorption curve, as shown in Figure 3a. Notably, the hysteresis loop is not apparent when the relative pressure is 0.45–1.0, suggesting the dominant presence of micropores [28]. The isotherm of AC450-4:8:2 displayed a characteristic type IV curve with an apparent hysteretic loop, indicating many mesoporous structures. Additionally, at higher relative pressure (P/P0 > 0.8), the curve exhibited a trailing phenomenon, indicating that the adsorption capacity increased significantly, which was caused by the capillary condensation of large pores. The adsorption isotherms of AC450-4:8:4 also exhibited similar characteristics, indicating the coexistence of micropores, mesoporous pores, and macropores. The pore size distribution in Figure 3b also confirmed the above conclusion. After adding NaHCO_3_ as an activator, the mesoporous volume (V_meso_) of HPCs significantly increased, while the original micropore volume (V_micro_) decreased significantly. Table 1 details the S_BET_ and pore size parameters of the three activated carbons. The S_BET_ of HPCs gradually decreased from 1017.1 m^2^/g to 553.9 m^2^/g after adding NaHCO_3_, along with a corresponding reduction in V_micro_ from 0.409 mL/g to 0.229 mL/g. The decline in micropores could be attributed to the transformation of micropores into meso- and macropores under the influence of NaHCO_3_. This was also demonstrated by the reduction of the honeycomb porous structure on the surface of AC450-4:8:0 in the SEM image (Figure 2b,d). After co-activation, there was little change in the total pore volume (V_Total_) of HPCs. But the mesoporous ratio (V_meso_/V_Total_) increased from 32.4% to 65.1%, and the average pore size (D_pore_) increased from 2.34 nm to 4.74 nm, which was attributed to the release of gas and the etching of sodium ions during the activation of NaHCO_3_.

The impact of carbonization temperature on the activation of ZnCl_2_ alone and the co-activation of NaHCO_3_ with ZnCl_2_ was further investigated. The N_2_ adsorption and desorption curves (Figure S5) and pore structure data (Table S1) are presented. In the temperature range of 400 to 600 °C, the activation temperature has limited influence on the S_BET_ of both HPCs, stabilizing at approximately 1000 m^2^/g and 700 m^2^/g, respectively. The V_meso_ of ACT-4:8:0 gradually increased from 0.147 mL/g to 0.342 mL/g upon ZnCl_2_ activation, between temperatures of 400 °C and 550 °C, while the V_micro_ remained stable at around 0.408 mL/g. On the other hand, employing the co-activation method generally resulted in higher V_meso_ values for ACT-4:8:2 compared to ACT-4:8:0, with a slowly increasing trend observed for V_micro_, which remained lower than that of ACT-4:8:0. At the carbonization temperature of 600 °C, there was a significant decrease in S_BET_, V_meso_, and V_meso_ of HPCs, indicating pronounced pore collapse. In conclusion, the promotion effect of ZnCl_2_ activation on micropores increases with temperatures ranging from 400 °C to 600 °C. However, it should be noted that higher temperatures resulted in the collapse of micropores and subsequently led to an increase in V_meso_. On the other hand, adding NaHCO_3_ facilitates mesoporous pores’ formation while inhibiting micropores’ formation.

#### 3.1.3. Analysis of Graphitization Degree

**Figure 4:**
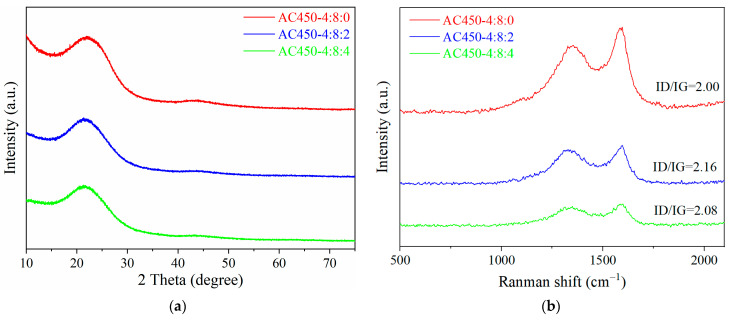
XRD (**a**) and Raman (**b**) spectra of AC450-4:8:0, AC450-4:8:2, and AC45-4:8:4.

Figure 4a shows the X-ray diffraction analysis of AC450-4:8:0, AC450-4:8:2, and AC450-4:8:4, and two diffraction peaks can be observed. The diffraction peak near 2θ = 22° corresponds to the (002) reflection of graphite, and the weak diffraction peak near 2θ = 44° corresponds to the (100) reflection of graphite. The two irregular humps indicate that the prepared activated carbon sample is an amorphous carbonaceous material composed of irregularly arranged aromatic carbon sheets [29]. After adding NaHCO_3_ as the activator, the intensity of two diffraction peaks of AC450-4:8:2 and AC450-4:8:4 decreased, indicating that the graphitization degree of the sample decreased [16]. As shown in Figure 4b, the same conclusion can be obtained from Raman spectroscopy. The G-band at 1603 cm^−1^ indicates in-plane vibration of aromatic carbon atoms, while the D-band at 1343 cm^−1^ is attributed to disordered or defective carbon structures [30]. The ratio of D-band to G-band intensity (ID/IG) can characterize the degree of defect of the carbon material [31]. The ID/IG values of AC450-4:8:2 and AC450-4:8:4 were overtly increased compared to AC450-4:8:0 (IG/IG = 2.00), with respective values of 2.16 and 2.08. This indicates that after adding NaHCO_3_ during activation, the graphitization degree of the sample was significantly reduced and the defects of the carbon material were increased due to the etching effect of alkaline metal ions [13].

#### 3.1.4. Analysis of Trace Elements and Surface Functional Groups

**Figure 5:**
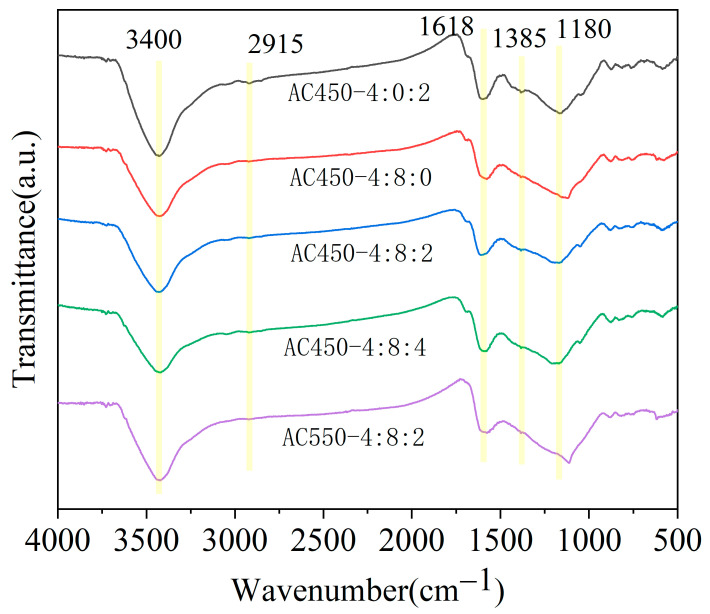
FT-IR spectra of samples under different activation conditions.

The surface functional groups of the samples were analyzed using FT-IR. Figure 5 shows that all solid samples obtained under different modified conditions exhibit a broad band around 3400 cm^−1^ corresponding to the stretching vibration of O-H in alcohols and phenols. The small peak near 1915 cm^−1^ is due to the stretching vibration of the C-H bond. The absorption peak of 1618 cm^−1^ is due to the stretching vibration of the aromatic ring C=C. The absorption peak of 883 cm^−1^ is due to the out-of-plane bending vibration of C=C in the phenyl group. The intensity of various characteristic peaks of different samples did not show much difference, and the difference was that the distribution of C-O stretching vibration peaks in the range of 1000–1300 cm^−1^ was other. The absorption peaks at 1050 cm^−1^ and 1080 cm^−1^ were observed in the AC450-4:0:2, AC450-4:8:2, and AC450-4:8:4 samples modified by NaHCO_3_ at 450 °C, while only appearing at 1100 cm^−1^ in the AC450-4:8:0 and AC550-4:8:0 samples. This was because different modification methods have different effects on the distribution of oxygen functional groups of catalyst precursors, resulting in other distributions of C-O bond stretching vibration peaks between 1000 cm^−1^ and 1300 cm^−1^. Due to the similarity of C-O stretching vibration in different chemical environments and the limitation of infrared spectroscopy testing, accurately determining the cause of this difference is impossible. Therefore, XPS was utilized to analyze the C and O surface configurations on all samples and perform quantitative analysis of various functional groups.

**Figure 6:**
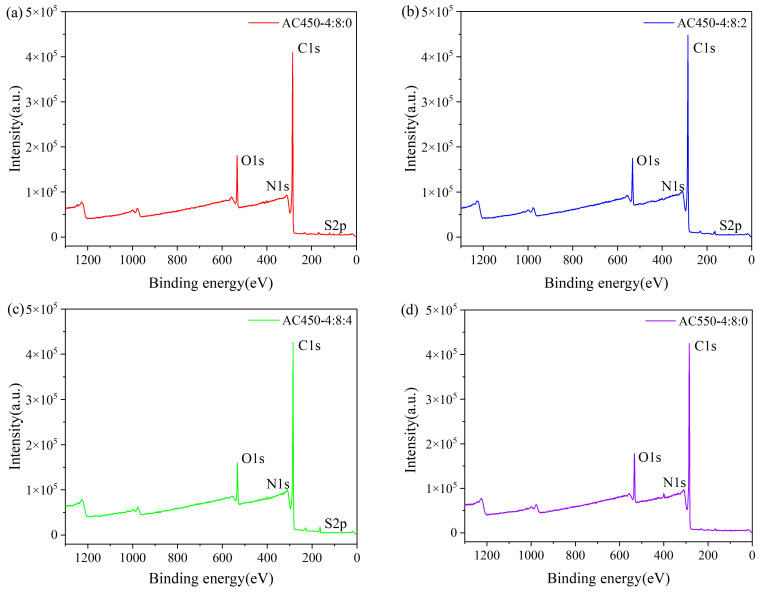
X-ray photoelectron spectroscopy (XPS) spectra of (**a**) AC-450-4:8:0, (**b**) AC450-4:8:2, (**c**) AC-450-4:8:4, and (**d**) AC550-4:8:2.

Figure 6 shows the XPS spectra of different samples. The surface element content of different samples is similar. The difference is that no apparent S content is detected in the AC550-4:8:2, indicating that the S element is not steadily doped into the aromatic ring structure during the carbonization process of LS [32]. The influence of S on the physicochemical characteristics of HPCs is limited and not further investigated in this study. Table 2 presents the semi-quantitative results of surface elements for all samples. With the addition of NaHCO_3_, the content of the C element increased, and the content of the O element decreased. This is because, at the activation temperature of 450 °C, sodium ions chelate with hydroxyl and ether groups and promote the cross-linking of carbon through the dehydration reaction [14].

**Figure 7:**
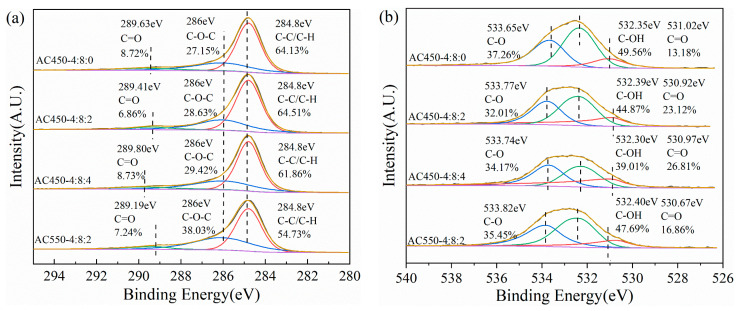
C1s (**a**) and O1s (**b**) X-ray photoelectron spectroscopy (XPS) spectra obtained for HPCs.

Figure 7 shows high-resolution XPS scans of C1s and O1s. In the high-resolution C1s spectrum, peaks in binding energies near 284.8 eV, 286.0 eV, and 289.5 eV appear, attributed to the sample’s C-C/C-H, C-O-C, and C=O, respectively. In the O1s spectrum, binding energy peaks near 531.0 eV, 532.3 eV, and 533.7 eV are observed, which are attributed to the C=O, C-OH, and -C-O of the sample, respectively. By comparing the XPS pattern of O1s before and after NaHCO_3_ modification, it was found that the C-OH functional group content of the modified sample decreased significantly. In contrast, the C=O group content increased (Table 3). It is speculated that this is due to the dehydration and oxidation of alkali metal ions during the activation process, which leads to the decrease of hydroxyl content and the increase of carboxylic acid content. As shown in Table 4, this can be confirmed by acid-base titration data of the sample. In conclusion, on the basis of ZnCl_2_ activation, the addition of NaHCO_3_ changes the content and distribution of oxygen functional groups on the surface of the sample. The stability and catalytic activity of the catalyst in the hydration reaction are greatly influenced by the oxygen-containing functional groups on the catalyst support. Existing research shows that show that hetero polyacid can achieve a stable load through hydrogen bonding with surface oxygen functional groups [33,34,35]. The increase of carboxylic acid functional groups is anticipated to enhance the cyclic stability of the catalyst, a hypothesis that has been substantiated in subsequent investigations. Additionally, previous research has demonstrated that augmenting the concentration of solid acid (carboxylic acid) in the catalyst promotes enhanced selectivity in the hydration reaction of α-terpineol [36].

### 3.2. Characterization and Catalytic Activity of Catalysts

#### 3.2.1. Characterization of Catalysts

**Figure 8:**
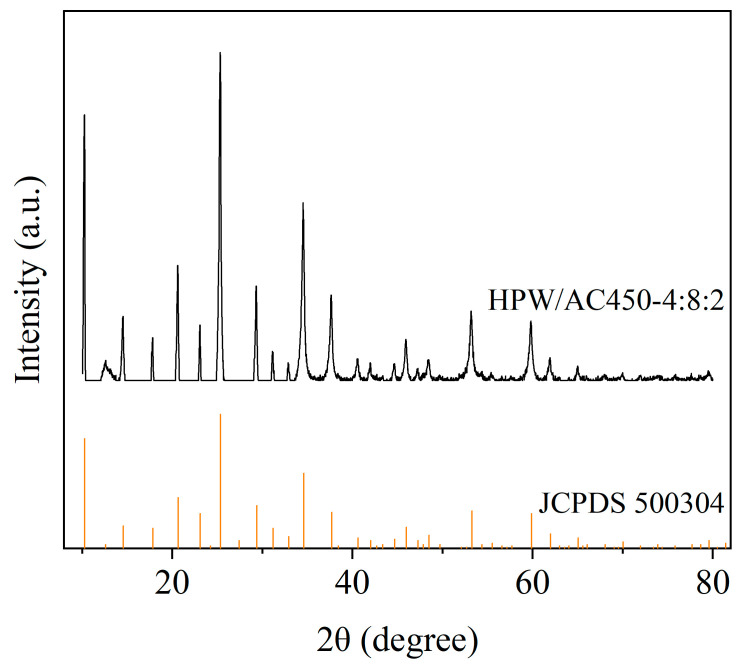
XRD patterns of HPW/AC450-4:8:2.

In order to verify whether phosphotungstic acid was successfully loaded on the HPCs surface, HPW/AC450-4:8:2 was characterized by XRD and SEM. The strong characteristic diffraction peak of H_3_PW_12_O_40_·6H_2_O (JCPDS 500304) was detected in the XRD pattern (Figure 8), indicating that phosphotungstic acid was successfully loaded on the HPCs surface in the form of hexahydrate. SEM images also showed that the surface of catalyst HPW/AC450-4:8:2 (Figure S6) was covered with a layer of white material, and no isolated HPW particles were observed. EDX spectroscopy enables qualitative analysis of diverse chemical elements in catalysts. The EDX spectrum of HPW/AC450-4:8:2 demonstrates the homogeneous distribution of carbon, oxygen, phosphorus, and tungsten within the catalyst matrix (Figure S7). SEM and EDX analysis revealed a uniform loading of HPW onto the HPCs surface.

Additionally, acid-base titration experiments revealed that the HPW/AC450-4:8:2 exhibited A_Total_ of 1.90 mmol/g and a strong acid value (A_HPW_) of 1.07 mmol/g. After calcination, the pure HPW displayed a considerable acid value of 2.12 mmol/g. Based on its preparation steps, the theoretical strong acid value of HPW/AC450-4:8:2 can be inferred to be 1.06 mmol/g. The experimental value is close to the theoretical value, indicating that the mass ratio of the support and phosphotungstic acid in the catalyst is comparable to 1:1.

#### 3.2.2. Hydration Reaction of α-Pinene

The one-step hydration reaction of α-pinene, catalyzed by a solid acid, proceeds via the protonation of α-pinene to form the pinanyl cation, followed by a ring-opening reaction yielding the p-menthenyl cation, and ultimately undergoes hydration to yield α-terpineol [37]. This process is accompanied by the generation of a large number of by-products due to the complexity of the reaction. For example, the pinanyl cation undergoes a series of rearrangements into borneol, camphene, etc., and the p-menthenyl cation removes a proton to from limonene, which is then rearranged into 1,3-cyclohexadiene [38,39]. Therefore, the preparation of suitable catalysts to reduce the formation of by-products and obtain higher α-terpineol yield has become the focus of research. Firstly, carbon-based solid acid catalysts need to have a large number of strong B-acidic sites in order to facilitate ring-opening reactions to increase the conversion of α-pinene. Secondly, the production of α-terpineol requires the participation of water molecules, which requires the material to have a certain hydrophilicity. Heteropolyacid is a kind of superacid, but its relatively low S_BET_ and high solubility in polar solvents result in its limited application in heterogeneous reactions [25]. When phosphotungstic acid was loaded on the porous carbon material with a high S_BET_, the accessibility of strong acid sites was increased, and the carbon material was endowed with certain hydrophilicity, which was conducive to the conversion of α-pinene molecules into α-terpineol on the catalyst surface.

#### 3.2.3. Catalytic Activity of Catalyst

**Figure 9:**
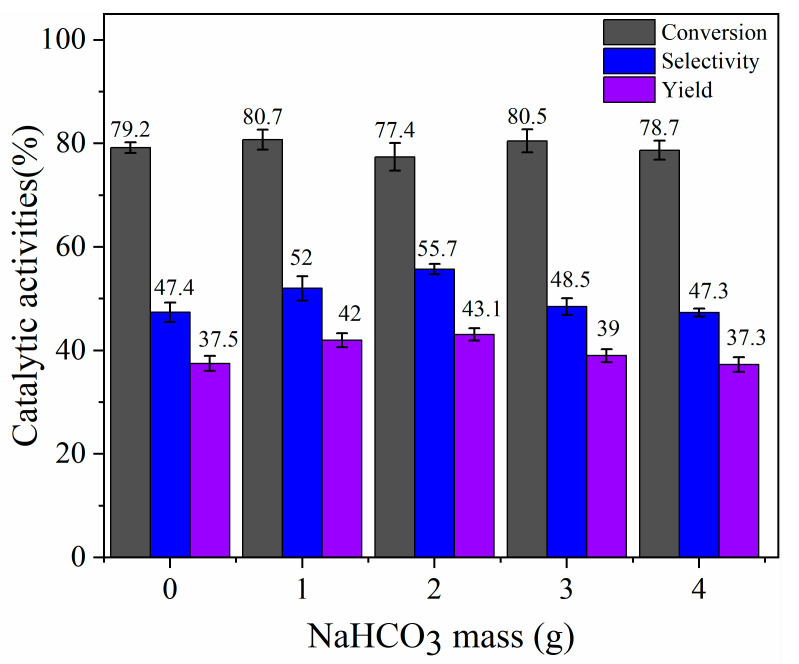
The catalytic hydration of α-pinene over catalysts prepared with different amounts of NaHCO_3_.

In order to study the effect of the amount of activator NaHCO_3_ (0.00–4.00 g) on the catalytic activity of carbon-based solid acid based on ZnCl_2_ activation, different catalysts were prepared for the hydration reaction. As shown in Figure 9, when only ZnCl_2_ was activated, the conversion rate of α-pinene was 79.2%, and the selectivity and yield of α-terpineol were 47.4% and 37.5%, respectively. With the increase in the amount of NaHCO_3_, the conversion of α-pinene stabilized at about 79.0%, and the selectivity of α-terpineol increased first and then decreased. When the dosage of NaHCO_3_ was 2 g, the yield of α-terpineol reached 43.1%. The vertical and sectional diameters of α-pinene were 0.84 nm and 0.77 nm, respectively, while those of α-terpineol were 1.18 nm and 1.46 nm, respectively [40]. After modification with NaHCO_3_, the average pore diameter of the carrier was increased from 2.34 nm to 4.35 nm. It is reasonable to assume that the increased selectivity of α-terpineol is due to the suitable pore size reducing the steric hindrance of α-terpineol formation while also ensuring that the target product α-terpineol can easily escape from the catalyst. In addition, compared with the sulfonated biochar solid acid catalyst (97.1% and 46.8%, respectively), the catalyst prepared in this study has a relatively low conversion rate and high selectivity, which is attributed to the high hydrophilic ability of heteropolyacid, which improves the accessibility of water molecules but reduces the affinity of solid acid catalyst for α-pinene [36].

**Figure 10:**
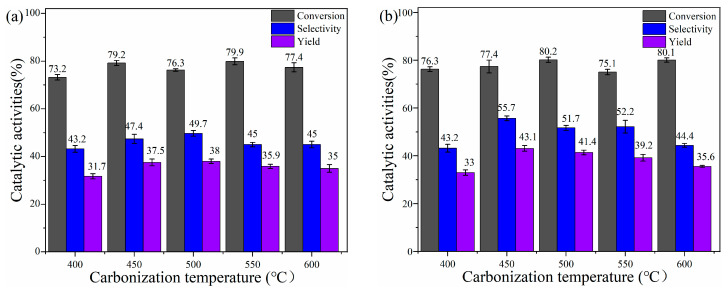
The catalytic hydration of α-pinene over catalysts by ZnCl_2_ (**a**) and the mixture of ZnCl_2_ and NaHCO_3_ (**b**) activation at different temperatures.

The literature shows that ZnCl_2_ has the best activation effect at 400–600 °C, so this study only investigated the catalytic activity of catalysts prepared in this temperature range [7]. Figure 10b shows the conversion and selectivity of the hydration reaction with catalysts prepared with the same lignin, ZnCl_2_, and NaHCO_3_ mass ratio (4:8:2) and different activation temperatures. The conversion of α-pinene remained relatively stable as the temperature increased, while the selectivity of α-terpineol exhibited an initial increase followed by a decrease. Notably, the maximum hydration yield of 43.1% was achieved at 450 °C. The hydration data of the catalyst activated only ZnCl_2_ also showed the same pattern, and the overall yield decreased significantly, indicating that the catalytic activity of the catalyst in the hydration reaction was improved considerably after adding the activator NaHCO_3_.

**Figure 11:**
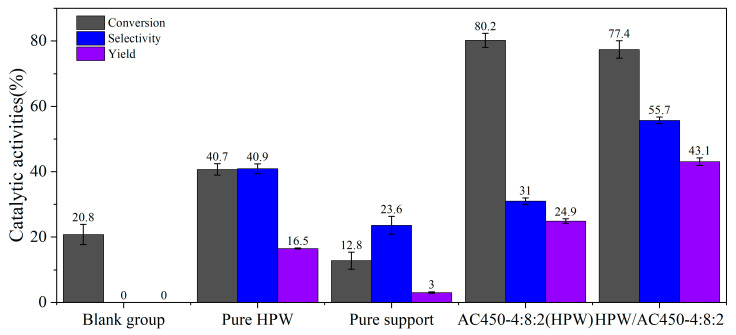
Hydration reactivity of catalyst under controlled experiment.

Furthermore, a series of controlled experiments were conducted to validate the catalytic activity of the catalyst synthesized in this study further. As depicted in Figure 11, α-terpineol production was not detected in the blank experiment. The pure support and pure HPW exhibited lower catalytic activity, resulting in α-terpineol yields of 16.5% and 3.0%, respectively, under identical hydration conditions. The absence of strong acid sites on the activated carbon carrier and the high solubility of pure HPW in the water phase contribute to this phenomenon. Using a physical mixture of AC450-4:8:2 and HPW with a mass ratio of 1:1 for the hydration reaction, AC450-4:8:2 (HPW) exhibited a conversion rate of 80.17%, selectivity of 31.0%, and yield of 24.9%. In this study, the selectivity for the hydration reaction was enhanced to 55.7% by employing an HPW/AC450-4:8:2 catalyst, indicating successful loading of HPW onto the HPCs surface. The improved catalyst selectivity can be attributed to the synergistic effect between strong acidic active sites and abundant oxygen functional groups on activated carbon’s surface, facilitating the stabilization of intermediate products during the hydration reaction [36].

To assess the catalyst’s repeatability, three consecutive catalytic experiments were conducted under identical conditions to determine the service life of HPW/AC450-4:8:0 and HPW/AC450-4:8:2. As depicted in Table 5, the conversion and selectivity of the hydration reaction decrease with an increasing number of cycles, resulting in a yield below 10% after three cycles. Furthermore, it could be observed that the decline in catalytic performance of NaHCO_3_ was mitigated following modification. The existing literature suggests that a significant number of micropores can aid in preventing active site loss and enhancing catalyst cycle stability due to their small size [41]. However, this study revealed that the cyclic strength of the catalyst did not diminish after NaHCO_3_ modification; instead, it exhibited an increase. This enhancement could be attributed to the steric hindrance effect of the hydration reaction, leading to a concentration of effective reaction sites within the mesoporous region. Additionally, the Raman spectrum analysis revealed a noticeable increase in the degree of defect in HPCs after NaHCO_3_ participated in the modification. This site could be an anchor point for stabilizing metal atoms, enhancing catalytic performance stability [42]. The catalyst’s strong acid content (A_HPW_) was measured through acid-base titration before the hydration reaction to investigate the cause of the decline in catalyst activity during the cyclic experiment. HPW/AC450-4:8:2 showed a 23.4% reduction in strong acid after one hydration experiment. Considering the non-negligible mass loss of the catalyst during the cycle experiment, this decrease in strong acid can be attributed to phosphotungstic acid leaching. Water involvement and a reaction time of up to 24 h are key factors contributing to the substantial leaching of phosphotungstic acid during the hydration reaction.

## 4. Discussion

The catalytic properties of the catalysts in this study and significant published results in the field of α-pinene hydration for α-terpineol production are summarized in Table 6 According to the existing literature, homogeneous catalysts exhibit evident catalytic advantages and find widespread applications in industry. However, they also raise environmental concerns and pose challenges regarding separation and recovery processes. The sulfonated carbon in the hydration reaction has exhibited exceptional catalytic performance in numerous studies. However, the inherent high-temperature sulfonation process poses challenges, such as environmental pollution and equipment corrosion, impeding heterogeneous catalysis application. The HPW/AC450-4:8:2 heterogeneous catalyst employed in this study exhibits high selectivity towards α-terpineol under relatively mild loading conditions of HPW. The statement above presents a novel perspective on investigating the application of carbon-based solid acid catalysts in the α-pinene hydration reaction.

## 5. Conclusions

Using sodium lignosulfonate as feedstock, the present study demonstrates the facile one-pot synthesis of hierarchical porous carbons (HPCs) with a rich pore structure through the co-activation strategy employing ZnCl_2_ and NaHCO_3_ at low temperatures. The results showed that with the addition of NaHCO_3_, the microporous structure of HPCs changed to mesoporous and macroporous, and the mesoporous ratio increased from 47.7% to 77.8%. This is attributed to the large amount of CO_2_ released by NaHCO_3_ in a two-step reaction during activation. Additionally, in comparison to the sole activation of ZnCl_2_, the co-activation strategy yields highly carboxylic acid-enriched and defect-rich HPCs. Finally, the solid acid catalyst was synthesized using HPCs as the carrier and HPW as the active site. The catalytic activity exploration reveals a significant enhancement in the hydration reaction selectivity of HPW/AC450-4:8:2 (55.7%), attributed to the synergistic effect between strong acid sites and carboxylic oxygen functional groups, thereby confirming successful loading of HPW. This study presents a relatively eco-friendly process for preparing HPCs at low temperatures, thereby contributing to reduced energy consumption and minimizing pollutant release (HCl) during ZnCl_2_ activation. Additionally, it offers a novel approach to enhancing the selectivity of hydration reactions.

## Figures and Tables

**Table 1:** Summary of the S_BET_, pore volume values, and pore size.

HPCs	S_BET_ (m^2^/g)	V_Total_ (mL/g)	V_micro_ (mL/g)	V_meso_ (mL/g)	V_meso_/V_Total_ (%)	D_pore_ (nm)
AC450-4:8:0	1017.1	0.595	0.402	0.193	32.4%	2.34
AC450-4:8:2	736.5	0.752	0.273	0.479	63.9%	4.35
AC450-4:8:4	553.9	0.656	0.229	0.427	65.1%	4.74

**Table 2:** Elemental contents of AC-450-4:8:0, AC450-4:8:2, AC-450-4:8:4, and AC550-4:8:2 determined by XPS.

HPCs	C1s (wt%)	O1s (wt%)	N1s (wt%)	S2p (wt%)
AC450-4:8:0	86.08	11.87	1.14	0.97
AC450-4:8:2	87.84	9.96	0.97	1.24
AC450-4:8:4	88.15	9.65	0.55	1.65
AC550-4:8:2	87.47	10.96	1.58	0

**Table 3:** Carbon and oxygen contents of HPCs by XPS.

HPCs	C (wt%)			O (wt%)		
	C-C/C-H	C-O-C	C=O	C=O	C-OH	-O-C
AC450-4:8:0	55.20	23.37	7.51	1.56	5.88	4.42
AC450-4:8:2	56.67	25.15	6.00	2.30	4.47	3.19
AC450-4:8:4	54.53	25.93	7.70	2.59	3.76	3.30
AC550-4:8:2	47.87	33.26	6.33	1.85	5.23	3.89

**Table 4:** Acid amount of HPCs.

HPCs	A_Total_ (mmol/g)	A_COOH_ (mmol/g)	A_OH_ (mmol/g)
AC450-4:8:0	0.83	0.37	0.46
AC450-4:8:2	0.93	0.56	0.37
AC450-4:8:4	0.86	0.46	0.40

**Table 5:** Reusability of catalyst.

Catalyst	Conversion Rate (%)	Selectivity (%)	Yield (%)	A_HPW_ (mmol/g)
HPW/AC450-4:8:2(1)	77.4	55.7	43.1	1.07
HPW/AC450-4:8:2(2)	53.6	38.2	20.5	0.82
HPW/AC450-4:8:2(3)	33.5	25.9	8.7	0.32
HPW/AC450-4:8:0(1)	79.2	47.4	37.5	1.08
HPW/AC450-4:8:0(2)	31.3	23.2	7.3	0.74
HPW/AC450-4:8:0(3)	29.4	18.7	5.5	0.26

**Table 6:** Comparison of catalytic properties of different catalysts for the synthesis of α-pinene into α-terpineol.

Entry	Catalyst	Conditions	Solvent	Conv. (%) α-Pinene	Selectivity (%)	Ref.
1	Ternary composite catalysts	α-pinene: water: acetic acid: tartaric acid: boric acid = 10:10:25:0.5:0.4, 60 °C, 24 h	-	96.1	58.7	[43]
2	IL	α-pinene: water: H_2_SO_4_ = 10.2:12:3, 70 °C, 4 h	-	93.2	26.8	[44]
3	Montmorillonite K10	α-pinene: water = 1:7.5, 80 °C, 24 h	1,4-dioxane	60	45	[45]
4	Sulfonated carbon	α-pinene: water = 1:1, 80 °C, 24 h	isopropanol	97.8	53.4	[36]
5	Sulfonated carbon	α-pinene: water = 1:1, 80 °C, 24 h	acetone	87.15	54.19	[40]
6	OA·2H2O/Bet	α-pinene: water = 1:5, 80 °C, 8 h	-	91.91	34.63	[46]
7	HPW/AC450-4:8:2	α-pinene: water = 1:1, 80 °C, 24 h	acetone	77.4	55.7	This work

## Data Availability

Data are contained within the article.

## Supplementary Materials

The following supporting information can be downloaded at: https://www.mdpi.com/article/10.3390/ma17040942/s1, Text S1: Products analysis; Text S2: Acid-base titration experiment; Figure S1: XRD patterns of the carbonized products of LS/activators compound precursors; Figure S2: Photo of HPC before pickling (a), AC250-4:8:2, (b) AC350-4:8:2 (c), and AC450-4:8:2; Figure S3: Photo of HPC before pickling (a), AC450-4:0:2, (b) AC450-4:8:0; Figure S4: SEM images of AC450-4:0:2; Figure S5: N_2_ adsorption and desorption isotherm of HPC (a) ACT-4:8:0, (b) ACT-4:8:2; Table S1: Summary of the surface area, pore volume values, and pore size; Figure S6: SEM image of HPW/AC450-4:8:2; Figure S7: EDX spectrum of HPW/AC450-4:8:2.
