# Supplementary material for: Investigation into the Structure and Properties of Biochar Co-Activated by ZnCl2 and NaHCO3 under Low Temperature Conditions
Source: Materials (Basel). 2024 Feb 18;17(4):942. doi: 10.3390/ma17040942 (PMC10890275; doi:10.3390/ma17040942)
Supplement: Supplementary file 1 [file materials-17-00942-s001.zip › materials-2838805-supplementary.pdf]

Supplementary

Investigation into the Structure and Properties of Biochar Co-Activated by $ZnCl_2$ and $NaHCO_3$ under Low Temperature Conditions

Hao Zhang [1], Zhaozhou Wei [1], Deyuan Xiong [1,2,*], Yao Wu [1], Menglong Tong [1], Huiping Su [1], Zuoyuan Zhang [1] and Jian Liao [1]

[1] School of Chemistry and Chemical Engineering, Guangxi University, Nanning 530004, PR China
[2] Guangxi Key Laboratory of Petrochemical Resource Processing and Process Intensification Technology, School of Chemistry and Chemical Engineering, Guangxi University, Nanning 530004, China
* Correspondence: dyxiong@gxu.edu.cn (D.X.); Tel.: +86-138-7718-2465 (D.X.)

Text S1. **Products analysis.**

All liquid products were analyzed using a gas chromatograph (GC-2010, Shimadzu, Japan) equipped with a polar capillary column (Rtx-Wax, RESTEK, USA, 30 m × 0.25 mm × 0.25 μm). The split ratio was 1:50, and nitrogen gas was the carrier gas. The gas chromatograph oven temperature was set at an initial temperature of 70°C, held for 10 min, increased at a rate of 5 °C/min to 150°C, and then increased to 240°C at a rate of 15 °C/min. The injector and detector temperatures were 240℃and 280℃, respectively. The reaction reactants and products were identified by comparing the retention times of α-pinene and α-terpineol standards. The conversion of α-pinene (X), the yield of α-terpineol (Y), and the selectivity (S) of α-terpineol were calculated according to the following equations:

$$X = \frac{m_{\alpha-pinene}}{m^0_{\alpha-pinene}} \times 100\% \tag{1}$$

$$Y = \frac{m_{\alpha-terpienol}}{\frac{M\alpha - terpienol}{M\alpha - pinene} \times m^0_{\alpha-pinene}} \times 100\% \tag{2}$$

$$S = \frac{Y}{X} \times 100\% \tag{3}$$

where $m^0_{\alpha\text{-pinene}}$ was the initially added volume of the α-pinene, $m_{\alpha\text{-pinene}}$ was the mass of α-pinene consumed (g), $m_{\alpha\text{-terpienol}}$ was the mass of α-terpienol (g). $M_{\alpha\text{-terpineol}}$ and $M_{\alpha\text{-pinene}}$ were the relative molecular mass of α-terpineol and α-pinene, respectively.

Text S2. **Acid-base titration experiment.**

Determination of Total Acid Value (A_{Total}) : The 0.25 g sample was accurately weighed using an analytical balance. Subsequently, 25 mL of a 0.05 mol/L NaOH standard solution was added to the weighed sample in a 100 mL beaker. The beaker was placed in a temperature-controlled ultrasonic cleaner at room temperature for one hour. After ultrasonic cleaning, the mixture was filtered, and the filtrate was collected in a conical flask. Methyl orange was added as an indicator, and titration was conducted using a 0.05 mol/L HCl standard solution. The titration was continued, with constant agitation, until the solution in the conical flask transitioned to a red colour. The volume of the consumed HCl standard solution was then recorded. The calculation formula for total acid value ATotal is as follows:

$$A_{Total} = \frac{0.05 \times 25 - 0.05x_1}{m} mmol/g \tag{4}$$

where A_{Total} represents the total acid value, unit mmol/g; x_1 represents the volume of HCl consumed, unit mL; and m represents the mass of the sample to be tested, unit g.

Determination of Carboxylic Acid Value (A_{-COOH}) of HPCs: A total of 0.25 g of the HPCs was accurately weighed using an analytical balance. Subsequently, 25 mL of a $NaHCO_3$ standard solution with a concentration of 0.05 mol/L was measured using a graduated cylinder. This solution was transferred to a 100 mL beaker, which was then placed in a CNC ultrasonic cleaner and subjected to ultrasonication at room temperature for one hour. Following ultrasonication, the solution was filtered, and the filtrate was collected in a conical flask. Methyl orange was introduced as an indicator, and the answer was titrated with a standard HCl solution of 0.05 mol/L concentration. The conical flask was agitated until the solution attained a red hue, and then the volume of the common HCl solution expended was recorded. The calculation formula for carboxylic acid value A_{-COOH} is as follows:

$$A_{-COOH} = \frac{0.05 \times 25 - 0.05x_2}{m} mmol/g \tag{5}$$

Among them, A_{-COOH} represents the carboxylic acid value, with a unit of mmol/g; x_2 represents the volume of HCl consumed, unit mL; and m represents the mass of HPCs to be tested, unit g.

Calculation of Weak Acid Content (A_{-OH}) of HPCs:

$$A_{-OH} = A_{Total} - A_{-COOH} \tag{6}$$

where A_{-OH} represents the weak acid content, unit mmol/g.

Calculation of Strong Acid Content (A_{HPW}) of HPW/AC450-4:8:2: A 0.25 g of catalyst was weighed precisely using an analytical balance. Then, 25 mL of a NaCl standard solution with a concentration of 0.05 mol/L was measured in a cylinder and transferred into a 100 mL beaker. Next, the beaker was placed in a CNC ultrasonic cleaner and sonicated for 1 h at room temperature. After filtering the cleaned sample, the filtrate was collected in a conical flask containing 25 ml 0.05 mol/L NaOH. Methyl orange was added as an indicator, and back-titration was conducted using a 0.05 mol/L HCl standard solution. The titration was continued, with constant agitation, until the solution in the conical flask transitioned to a red colour. The volume of the consumed HCl standard solution was then recorded. The calculation formula for total acid value A_{HPW} is as follows:

$$A_{HPW} = \frac{0.05 \times 25 - 0.05x_3}{m} mmol/g \tag{7}$$

where A_{HPW} represents the strong acid value, unit mmol/g; x_3 represents the volume of HCl consumed, unit mL; and m represents the mass of the sample to be tested, unit g.

Calculation of Strong Acid Content (A_{HPW}) of HPW after calcination: It is referred to the A_{Total} calculation method.

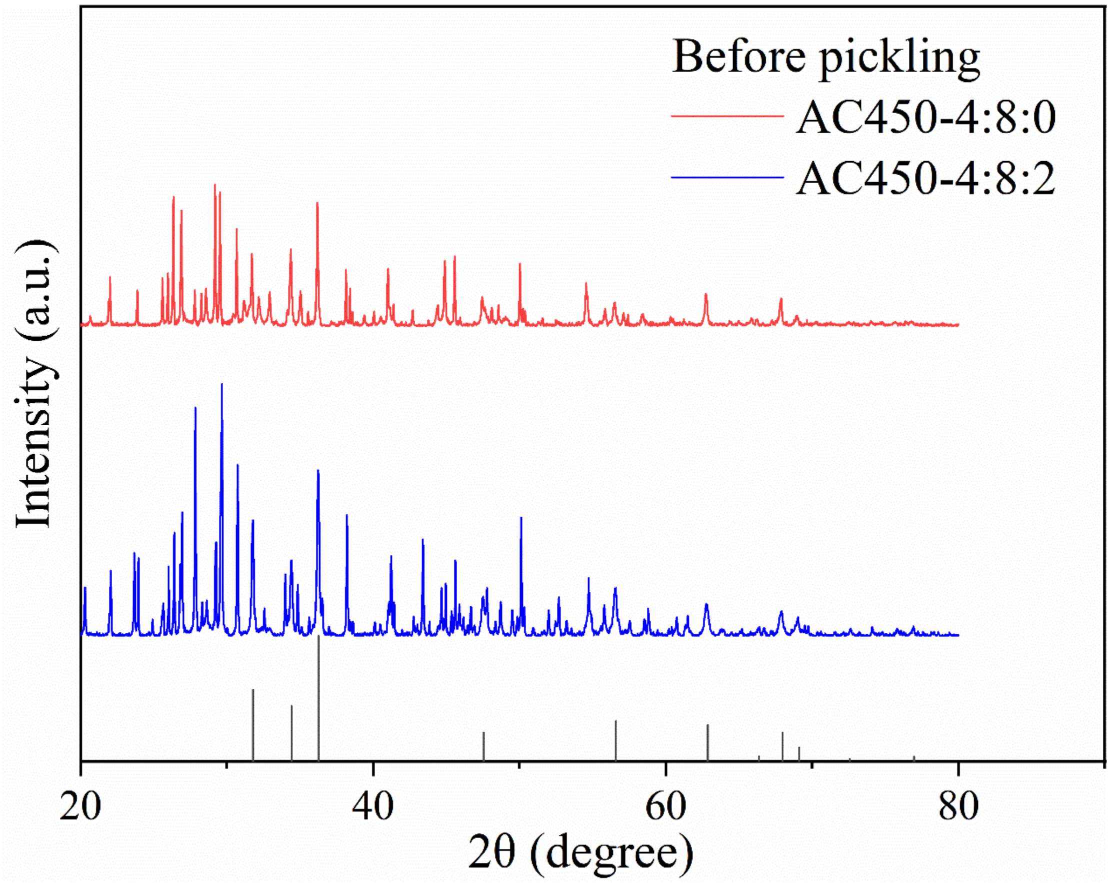

Figure S1. XRD patterns of the carbonized products of LS/activators compound precursors.

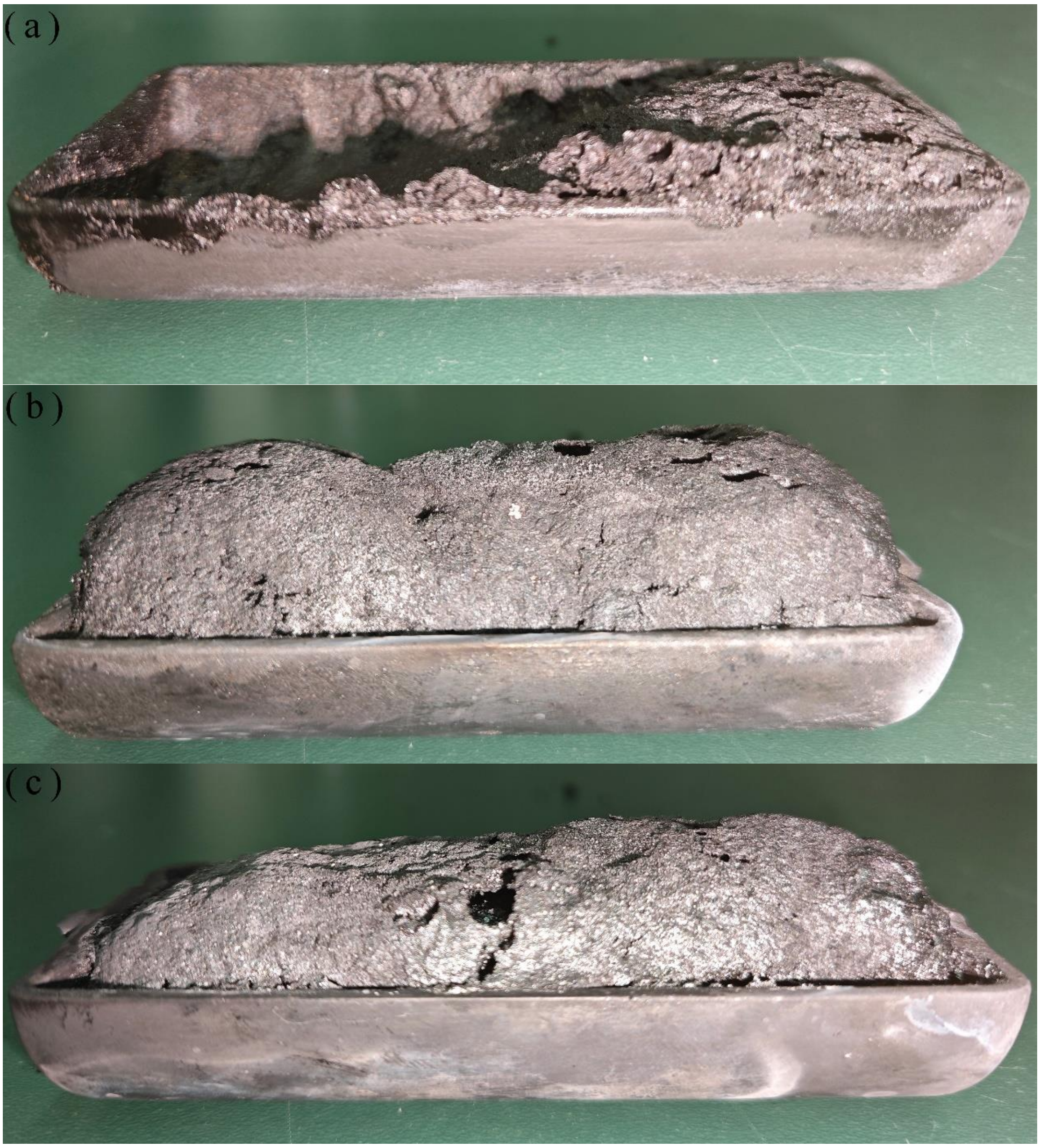

Figure S2. Photo of HPCs before pickling (a), AC250-4:8:2(b), AC350-4:8:2 (c), and AC450-4:8:2.

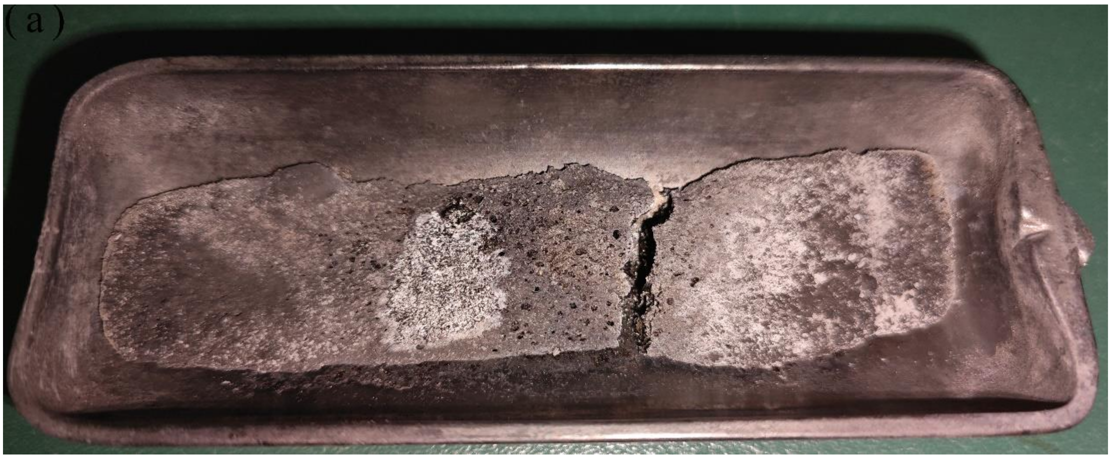

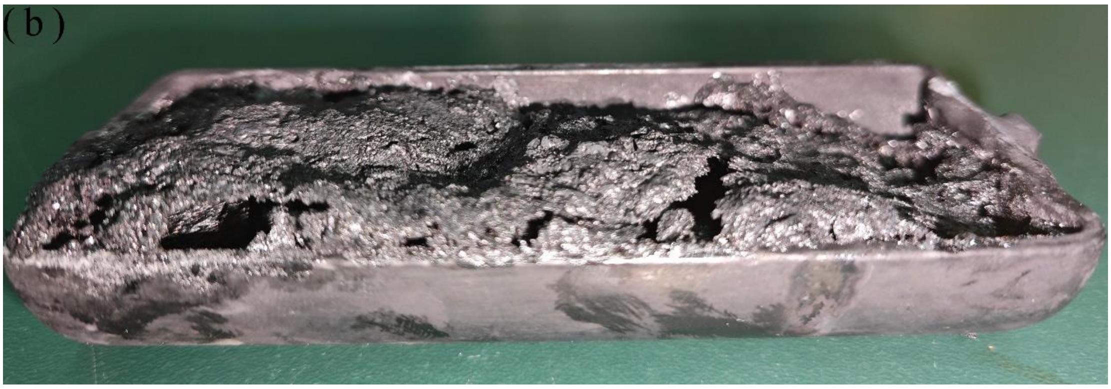

Figure S3. Photo of HPCs before pickling (a), AC450-4:0:2(b), AC450-4:8:0.

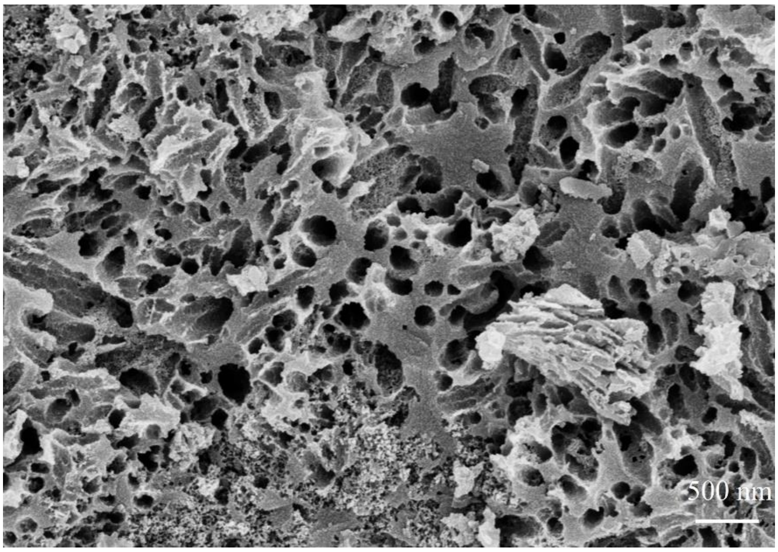

Figure S4. SEM images of AC450-4:0:2.

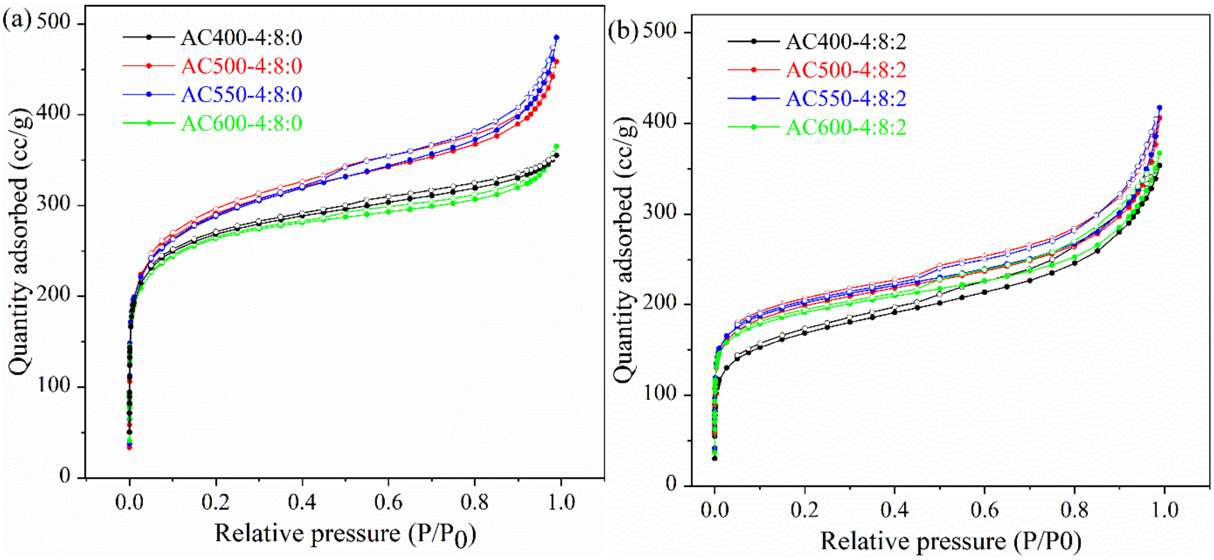

Figure S5. N_2 adsorption and desorption isotherm of HPCS (**a**) ACT-4:8:0, (**b**) ACT-4:8:2.

Table S1. Summary of the surface area, pore volume values, and pore size.

HPCs	S_{BET}(m²/g)	V_{total}(cc/g)	V_{micro}(cc/g)	V_{meso}(cc/g)	V_{meso}/V_{Total}(%)	D_{pore}(nm)
AC400-4:8:0	995.3	0.551	0.404	0.147	26.7%	2.21

AC450-4:8:0	1017.1	0.595	0.402	0.193	32.4%	2.34
AC500-4:8:0	1060.3	0.709	0.417	0.292	41.2%	2.68
AC550-4:8:0	1046.6	0.752	0.410	0.342	45.5%	2.87
AC600-4:8:0	972.3	0.565	0.381	0.184	32.6%	2.32
AC400-4:8:2	609.0	0.547	0.250	0.297	55.0%	3.59
AC450-4:8:2	736.5	0.752	0.273	0.479	63.7%	4.35
AC500-4:8:2	737.8	0.628	0.288	0.340	54.1%	3.29
AC550-4:8:2	746.8	0.647	0.294	0.354	54.7%	3.47
AC600-4:8:2	709.1	0.568	0.280	0.288	50.7%	3.20

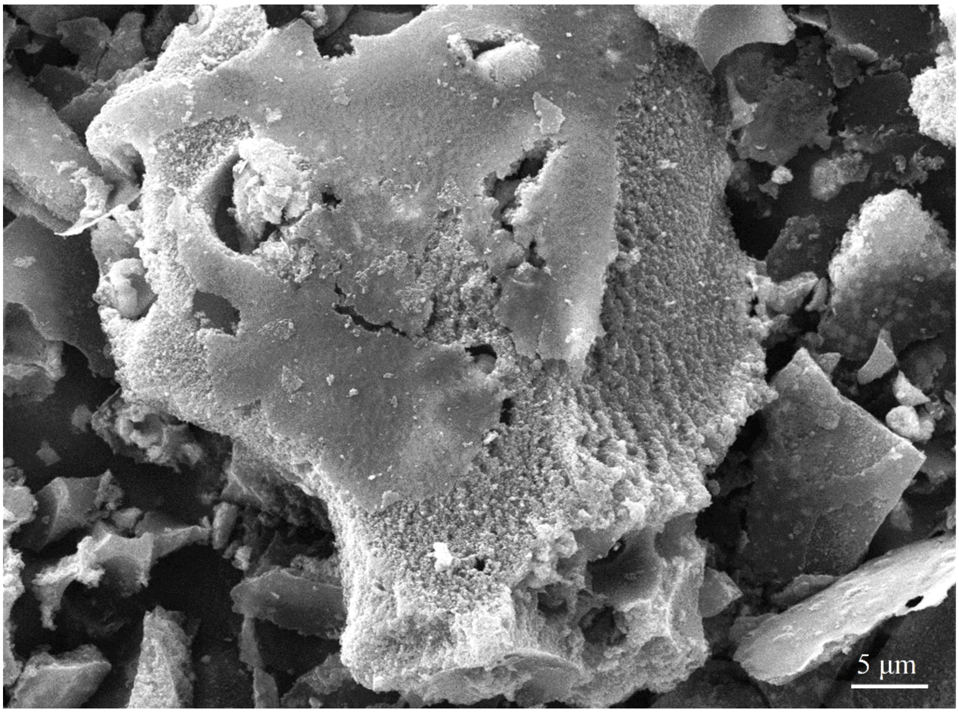

Figure S6. SEM image of HPW/AC450-4:8:2.

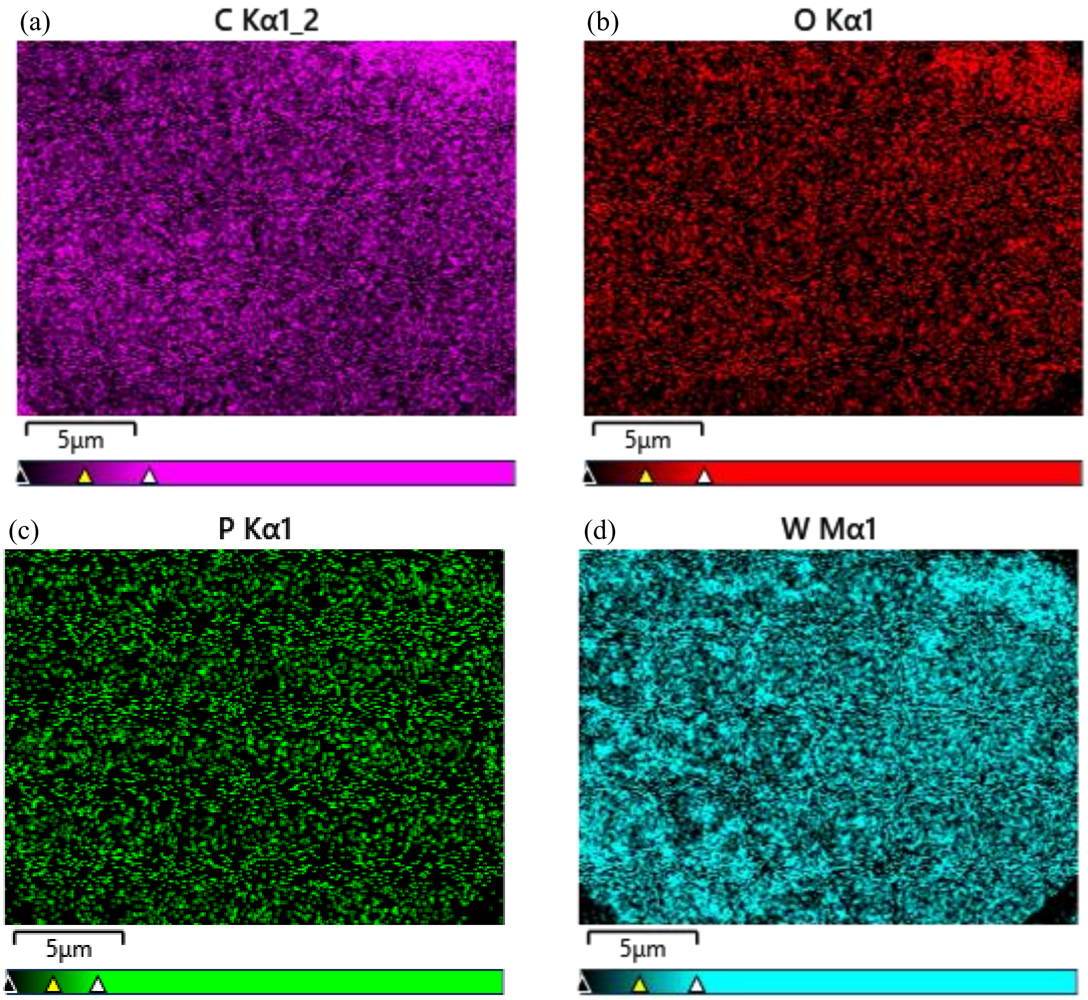

Figure S7. EDX spectrum of HPW/AC450-4:8:2.
